# Psychometric properties of the Caregiver’s inventory neuropsychological diagnosis dementia (CINDD) in mild cognitive impairment and dementia

**DOI:** 10.1007/s00702-023-02728-0

**Published:** 2024-01-10

**Authors:** Sofia Cuoco, Carlo Blundo, Monica Ricci, Arianna Cappiello, Rossella Bisogno, Immacolata Carotenuto, Anna Rosa Avallone, Roberto Erro, Maria Teresa Pellecchia, Marianna Amboni, Paolo Barone, Marina Picillo

**Affiliations:** 1 Department of Medicine, Surgery and Dentistry “Scuola Medica Salernitana”, Neuroscience section, Center for Neurodegenerative Diseases (CEMAND), University of Salerno, Via Allende, Baronissi, 84081 Salerno, Italy; 2 Department of Neuroscience, Center of Cognitive Disorders and Dementia, San Camillo Hospital, Rome, Italy; 3 IDC Hermitage-Capodimonte, Naples, Italy

**Keywords:** Behavioral disorders, Clinical interview, Cognitive disorders, Neuropsychological evaluation

## Abstract

**Objectives:**

The Caregiver’s Inventory Neuropsychological Diagnosis Dementia (CINDD) is an easy tool designed to quantify cognitive, behavioural and functional deficits of patients with cognitive impairment. Aim of the present study was to analyse the psychometric properties of the CINDD in Mild Cognitive Impairment (MCI) and Dementia (D).

**Design, setting and participants:**

The CINDD, composed by 9 sub-domains, was administered to fifty-six caregivers of patients with different types of dementia (D) and 44 caregivers of patients with MCI. All patients underwent an extensive neuropsychological assessment, the Neuropsychiatric Inventory (NPI) and functional autonomy scales. The reliability, convergent construct validity and possible cut-off of CINND were measured by Cronbach’s alpha (α), Pearson’s correlation and ROC analysis, respectively.

**Results:**

The D and MCI patients differed only for age (p=0.006). The internal consistency of CINDD was high (α= 0.969). The α-value for each CINDD domain was considered acceptable, except the mood domain (α=0.209). The CINDD total score correlated with cognitive screening tests; each domain of the CINDD correlated with the corresponding score from either tests or NPI (p<0.05), except for visuo-spatial perception skills and apathy. A screening cut-off equal to 59, can be used discriminate D from MCI (Sensitivity=0.70, Specificity=0.57).

**Conclusion:**

The CINDD is a feasible, accurate and reliable tool for the assessment of cognitive and behavioural difficulties in patients with different degree of cognitive impairment. It may be used to quantify and monitor caregiver-reported ecological data in both clinical and research settings.

**Supplementary Information:**

The online version contains supplementary material available at 10.1007/s00702-023-02728-0.

## Introduction

Cognitive decline and behavioral dysfunction and consequent impact on customary functional daily activities represent core features of the definition of dementia (APA, 2013). A number of clinical entities present a differential involvement of cognitive domains with memory being affected more frequently in Alzheimer's dementia (AD) (Albert et al. 2011), executive function and attention in frontotemporal dementia spectrum (FTD) (Boeve et al. 2022), and visuospatial functions in Lewy Body dementia (DLB) (Sanford 2018). Dementia is preceded by Mild Cognitive Impairment (MCI), a prodromal stage characterized by cognitive dysfunction not interfering with activities of daily living (Anderson 2019).

According to current recommendations (DSM-5), a detailed neuropsychological battery including objective neuropsychological standardized tests of attention, language, visuospatial perception, memory, and executive functions is warranted to diagnose dementia and MCI (APA, 2013). In real-world settings, a skilled neuropsychologist performs a clinical interview to investigate patients’ status and collect information from caregivers on functioning in daily life (Halligan et al. 2003; Mondini et al. 2003; Lezak et al. 2004; Snyder et al. 2006; Hebben and Milberg 2009; Schoenberg and Scott 2011). During the interview with both the patient and the caregiver, several aspects are reviewed including medical conditions, family history of dementia or other neurodegenerative diseases, and the impact of cognitive and behavioral deficit on daily activities.

The role of caregiver in reporting information on cognitive status and the relative degree of interference on activities of daily living is paramount. As such, the preliminary caregiver interview on cognitive status represents a key part of the neuropsychological formal exam extensively described in a number of manuals of clinical neuropsychology (Halligan et al. 2003; Hebben and Milberg 2009; Lezak et al. 2004; Snyder et al. 2006). However, there is a dearth of standardized scales possibly representing reliable surrogates for such purpose. Abbate et al. (2021) analyzed the psychometric properties of the NeuroPsychological Examination, a systematic collection of cognitive data based on the observation of the patient’s behaviors in the context of a clinical interview. The NeuroPsychological Examination is a flexible instrument, run through a clinical conversation with open questions. Complementary, Giordano et al. (2022) proposed the psychometric validation of the Cognitive Assessment Interview. The Cognitive Assessment Interview is a semi-structured interview with 10 items investigating cognitive domains and social cognition (Giordano et al. 2022). However, both such instruments have several limitations restricting their application on large scale in real-world settings.

The aim of the present work are to (1) propose an easy tool for screening cognitive and behavioral skills and disease interference on daily life applicable in real-world clinical settings and (2) verify its psychometric properties in comparison with well-established rating scales. The target of such a tool is the caregiver.

## Methods

### Description of Caregiver’s inventory neuropsychological diagnosis dementia (CINDD)

The Caregiver’s Inventory Neuropsychological Diagnosis Dementia (CINDD) scale is a tool developed by CB and MR to easily screen cognitive and behavioral skills and disease interference on daily activities also useful to choose the appropriate cognitive tests to be administered. The CINND is composed by 9 domains including memory (5 items), perceptive-spatial and praxis skills (10 items), language (8 items), executive functions (6 items), personality and social behavior (14 items), ideation/perception (6 items), mood (2 items), anxiety (2 items), and impact on activities of daily living (8 items). The questions refer to changes that occurred after the onset of the disease regarding the last 30–60 days. The caregiver is asked to mark with a cross the cell corresponding to the score relating to the severity/frequency of the symptom. The answers are presented on a Likert scale, where a score of zero corresponds to absent, a score of 1 to a mild or rare symptom, a score of 2 to a moderate or occasional symptom, and a score of 3 to a severe or frequent symptom. The total score is obtained from the sum of all the scores of the individual domains. The lowest score is zero and the highest score is 183. Higher scores correspond to worse cognitive-behavioral dysfunction. More specifically, the maximum score for the memory domain is 15, 30 for the perceptive-spatial and praxis skills domain, 24 for language, 18 for executive functions, 42 for the personality and social behavior domain, 18 for ideation/perception, 6 for mood, 6 for anxiety, and 24 for impact on activities of daily living.

The CINDD was created in Italian and translated into English by a native English speaker (SA) who is fluent in Italian. There were no significant differences between the two versions.

Both the Italian and English versions of the CINDD are reported in Appendix_1.

### Patients

Fifty-six patients diagnosed with D, 44 participants with MCI, and the corresponding 100 caregivers were enrolled consecutively and invited to participate to this study. Participants with severe dementia were excluded because they were not able to support a complete neuropsychological battery. The project was performed in accordance with the ethical standards laid down in the 1964 Declaration of Helsinki and was approved by the local Ethics Committee.

Specifically, the dementia cohort included 28 patients with AD, 19 within the FTD spectrum (14 with behavioral variant and 5 with language variant) and 9 with DLB. All qualified with the corresponding current clinical criteria for these diagnostic entities. The MCI cohort included 44 patients satisfying current clinical criteria (Petersen et al. 1999; Nelson and O’Connor 2008). All participants were screened with both the Mini Mental State Examination (MMSE) and the Montreal Cognitive Assessment (MoCA) and completed an extensive neuropsychological battery investigating four cognitive domains (memory, executive function, language, and visual-spatial skills) and including the Rey’s auditory 15-word learning test (RAVLT), the constructional apraxia task (CAT), the Benton Judgment of Lines Orientation Task (BJLOT), the phonemic and semantic fluency tests, the Rey–Osterrieth Complex Figure Test (ROCF), and the SAND naming subtest (Picillo et al. 2019a, b; Picillo et al. 2019a, b). Besides the CINDD, the caregivers completed the Activities of Daily Living (ADL) (Katz et al. 1970), the instrumental activities of daily living (IADL) (Lawton and Brody 1988), and the Neuropsychiatric Inventory (NPI) (Cummings et al. 1994). Moreover, caregivers, thinking about themselves, completed the 14-item Resilience Scale (RS-14) (Cuoco et al. 2022), the EuroQol- 5 Dimension (EQ-5D), the Visual Analog Scale (VAS) (Balestroni and Bertolotti 2012), and the Beck Depression Inventory Second Edition (BDI-II) (Wang and Gorenstein 2013).

### Statistical analysis

Normal distribution of variables was checked with the Kolmogorov–Smirnov test.

The following psychometric properties were explored for the CINDD total score and sub-domains: acceptability, internal consistency, and construct validity. Acceptability was considered appropriate for each CINDD item if there were ≤ 5% of missing values and for the total score if there were ≤ 15% of the lowest and highest possible scores (floor and ceiling effect). Moreover, skewness of total (limits, − 1 to + 1) was determined. Internal consistency was evaluated by means of Cronbach’s alpha. A value ≥ 0.70 was considered acceptable. Scaling assumptions referring to the correct grouping of items and the appropriateness of their summed score were checked using corrected item-total correlation for CINDD (standard, ≥ 0.40).

Construct validity was explored with Pearson’s correlation between the CINDD and its domains and the other administered tests and scales investigating the same skill/feature. Namely, we correlated the MMSE and the MOCA total scores with the CINDD total score, the Rey’s 15-word test and Rey’s complex figure deferred test with the CINDD memory, the Benton’s and the Milan constructive apraxia tests with the CINDD perceptual-spatial praxis skills, the phonemic fluency test and the copying of the complex figure of Rey with the CINDD executive functions, and the semantic fluency and the SAND naming test with the CINDD language. We also correlated the CINDD personality/social behavior with each NPI item measuring apathy, euphoria, irritability, agitation, disinhibition, aberrant motor behavior and nutrition, the CINDD ideation/perception with each NPI item measuring hallucinations and delusions, the CINDD mood with each NPI item measuring depression and apathy, and the CINDD anxiety with the NPI item measuring anxiety. Finally, we correlated the CINND-impact on activities of daily living domain with the Activity of daily living (ADL) and the instrumental Activity of daily living (IADL). Correlations were considered strong with coefficients > 0.70 and moderate with a coefficients between 0.30 and 0.70.

After analyzing the demographic characteristics of caregivers dividing according to patients’ diagnosis, using the *T* test and *χ*^2^ test were necessary, we measured the Pearson’s correlations between these caregiver’ data and CINDD total score.

Subsequently, a ROC analysis was performed for the CINDD total score to identify the optimal cut-off to discriminate dementia from MCI. Sensitivity, specificity, positive predictive value (PPV) and negative predictive value (NPV), and diagnostic accuracy in comparison to clinical diagnosis were assessed at the best threshold for classification.

The *T* test or the ANOVA test, corrected for multiple comparisons with Bonferroni’s test, were used to compare continuous variables between cohorts.

All data, analysis code, and research materials are available upon request from the first author. All analyses were performed by SPSS for Windows, version 23.0. All statistical tests were two tailed, with *α* set at 0.05.

## Results

### Sample characteristics

Patients with D and with MCI presented similar socio-demographic features (*p* > 0.025, corrected for Bonferroni’s Test), except for age which was higher in MCI (D vs MCI, 70.73 ± 7.83 vs 74.93 ± 6.94, *p* = 0.006) (Table 1). Moreover, no significant differences were found for socio-demographic variables between AD, FTD, DLB, and MCI (Table 2).

**Table 1:** Socio-demographic features of patients with Dementia (D) and Mild Cognitive Impairment (MCI)

	D (*N* = 56)	MCI (*N* = 44)	*p*
Age	70.73 ± 7.83	74.93 ± 8.87	0.006*
Education	9.12 ± 4.62	9.59 ± 5.31	0.640
Disease duration	2.95 ± 1.98	2.03 ± 1.17	0.039
Sex (M%)	48.2%	45.5%	0.780

Statistically significant differences are indicated in bold

*D* dementia, *M* males, *MCI* mild cognitive impairment

*Corrected for multiple comparisons

**Table 2:** Socio-demographic features of patients with Alzheimer’s dementia (AD), Frontotemporal dementia (FTD), Lewy Body dementia (DLB) and Mild Cognitive Impairment (MCI)

	AD (*N* = 28)	FTD (*N* = 19)	LBD (*N* = 9)	MCI (*N* = 44)	*p*
Age	70.5 ± 8.87	70.31 ± 7.33	72.33 ± 5.63	74.93 ± 8.87	0.048
Education	9.14 ± 4.33	10.15 ± 5.24	6.88 ± 3.65	9.59 ± 5.31	0.409
Disease duration	3.09 ± 2.21	2.75 ± 1.98	3.00 ± 1.41	2.03 ± 1.17	0.221
Sex (M%)	42.9%	57.9%	44.4%	45.5%	0.762

Statistically significant differences are indicated in bold

*AD* Alzheimer’s dementia, *FTD* frontotemporal dementia, *LBD* Lewy body dementia, *M* males, *MCI* mild cognitive impairment, *N* number

*Significant results by post hoc analysis

### Acceptability

The mean (± SD) CINDD total score was 69.41 (± 40.33). One-hundred percent of data were totally computable, and there were no missing values.

In the entire sample, neither the ceiling nor the floor effects were observed for the CINDD total score (lowest possible score = 2, 1%; highest possible score = 168, 1%). The skewness of the CINDD was within the standard limits (CINDD total score = 0.448).

The Kolmogorov–Smirnov test showed the CINDD total score was normally distributed (*p* = 0.20).

### Reliability

Cronbach’s alpha (*α*) was 0.969, and thus, it was considered acceptable for internal consistency. No item improved Cronbach’s alpha if removed. Item CINDD correlation was ≥ 0.40 for all questions, except for the first item of anxiety domain (*r* = 0.25).

We found a high reliability for memory (*α* = 0.852), perceptive-spatial and praxis skills (α = 0.935), language (*α* = 0.921), executive skills (*α* = 0.842), personality and social behavior (α = 0.908), and ideation/perception (*α* = 0.842) domains. Also the impact on activities of daily living domain presented high reliability (*α* = 0.874), while a low reliability was found for anxiety (α = 0.633) and especially for mood domain (*α* = 0.209).

### Convergent construct validity

As for convergent construct validity, significant inverse correlations emerged between the CINDD total score and the MMSE and MOCA as well as between the each CINDD cognitive domain and the corresponding test exploring that domain, except for the test that investigates visual perception function (Table 3). Furthermore, the CINDD affective and behavior domains correlated with the specific items of the NPI, except for the sub-domain of apathy, as well as the CINDD domain on impact of activities of daily living with both the ADL and IADL (Table 4).

**Table 3:** Pearson’s correlation between the CINDD total score and domains and corresponding cognitive tests

	CINDD total score		CINDD memory		CINDD perceptive- spatial praxis skills		CINDD language		CINDD executive skills	
	*R*	*p*	*r*	*P*	*r*	*p*	*R*	*P*	*r*	*p*
MMSE	− 0.49	**0.000**								
MOCA	− 0.34	**0.001**								
RAVLT delayed recall			− 0.35	**0.002**						
ROCF delayed copy			− 0.43	**0.000**						
BJLOT					− 0.23	0.18				
CAT					− 0.39	**0.000**				
Semantic fluency test							− 0.44	**0.000**		
SAND naming							− 0.53	**0.000**		
ROCF immediate copy									− 0.36	**0.000**
Phonemic fluency test									− 0.22	**0.05**

Statistically significant correlations are indicated in bold

*BJLOT* Benton judgment of lines orientation task, *CAT* constructional apraxia task, *CINDD* Caregiver’s inventory neuropsychological diagnosis dementia, *MMSE* mini mental state examination, *MOCA* montreal cognitive assessment, *RAVLT* Rey’s auditory 15-word learning test, *ROCF* Rey–Osterrieth complex figure test, *SAND* screening for Aphasia in NeuroDegeneration

**Table 4:** Pearson’s correlation between the CINDD behavioral domains and corresponding items from the NPI as well as between the CINDD domain on impact on ADL and IADL

	CINDD Personality and social behavior		CINDD Ideation/perception		CINDD Mood		CINDD Anxiety		CINDD Impact on activities of daily living	
	*R*	*P*	*R*	*p*	*R*	*p*	*r*	*P*	*r*	*p*
NPI euphoria	0.23	**0.019**								
NPI irritability	0.54	**0.000**								
NPI agitation	0.50	**0.000**								
NPI disinhibition	0.49	**0.000**								
NPI aberrant motor behavior	0.41	**0.000**								
NPI eating disorders	0.46	**0.000**								
NPI hallucinations			0.44	**0.000**						
NPI delusions			0.36	**0.000**						
NPI depression					0.44	**0.000**				
NPI apathy					0.15	0.123				
NPI anxiety							0.30	**0.003**		
ADL									− 0.27	**0.007**
IADL									− 0.42	**0.000**

Statistically significant correlations are indicated in bold

*ADL* activities of daily living, *CINDD* Caregiver’s inventory neuropsychological diagnosis dementia, *IADL* instrumental activities of daily living, *NPI* neuropsychiatric inventory

No significant differences were reported for demographic variables of caregivers and there were no significant correlations among age, education, EQ-5D, VAS, RS-14, and BDI-II of caregivers and CINDD total score (*p* > 0.05) (Appendix_2).

### Determining the cut-off of the CINDD

**Figure 1:**
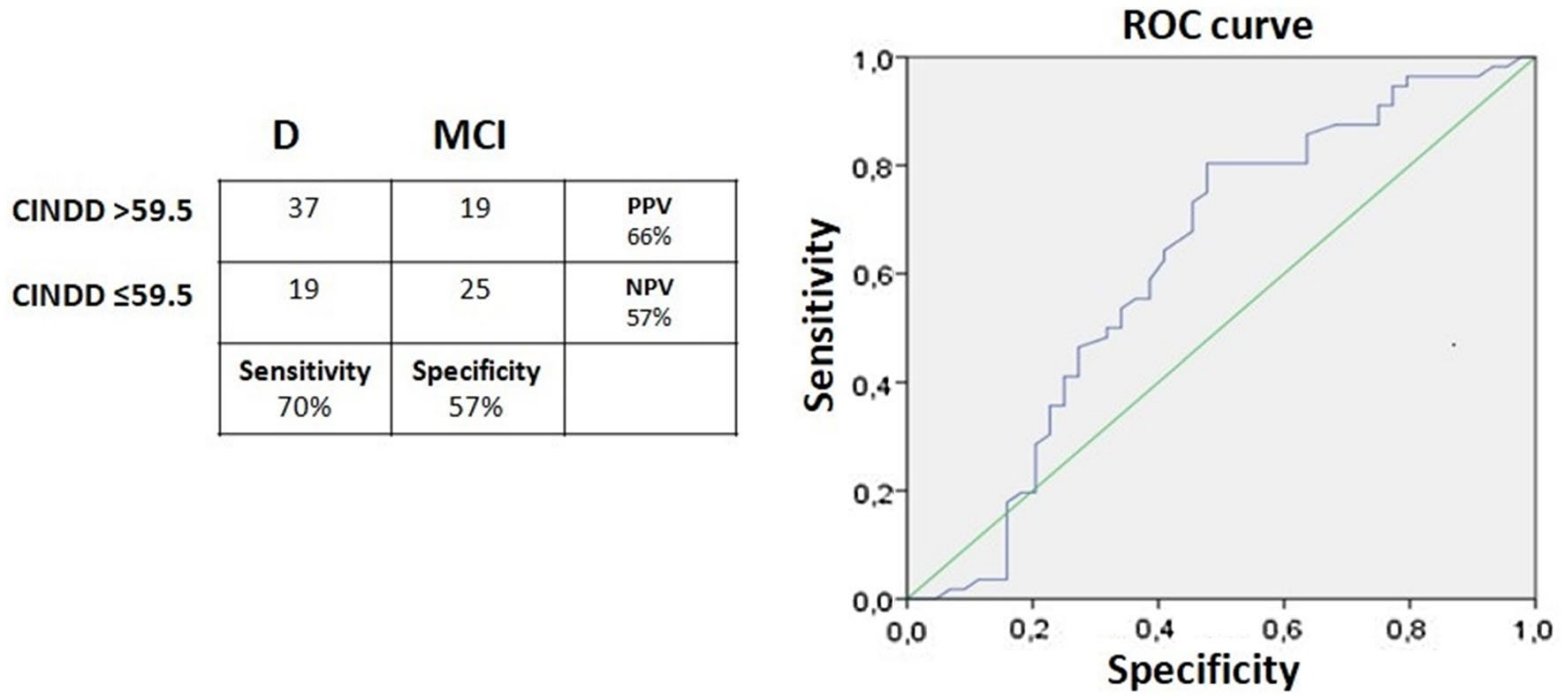
The ROC curve for discrimination of D and MCI with the CINDD total score. *CINDD* Caregiver’s inventory neuropsychological diagnosis dementia, *D* dementia, *MCI* mild cognitive impairment, *NPV* negative predictive value, *PPV* positive predictive value, *ROC* receiver operating characteristic

ROC analysis was used to assess the discriminatory power of the CINDD total score in discriminating between dementia and MCI. The determined cut-off was 59 showing 70% sensitivity, 57% specificity, 66% positive predictive value (PPV), 57% negative predictive value (NPV), and 62% diagnostic accuracy (Fig. 1).

The ROC analysis showed a 62% discriminatory power [95% confidence interval (CI), 51–74%].

Comparing the two groups determined with such cut-off, we found that patients who scored higher on the CINDD performed worse on screening cognitive tests as MMSE and MOCA, in executive functions, linguistic ability, and constructive apraxia tests and in activities of daily living (Table 5).

**Table 5:** Cognitive and behavioral features in two groups of patients identified with the CINDD cut-off of 59

	CINDD > 59	CINDD ≤ 59	*p*
MMSE	16.42 ± 7.03	22.60 ± 4.65	**0.000**
MOCA	10.81 ± 5.50	14.80 ± 5.46	**0.001**
RAVLT delayed recall	1.75 ± 2.51	2.45 ± 2.53	0.226
ROCF delayed copy	3.21 ± 4.10	4.36 ± 4.90	0.282
BJLOT	14.62 ± 3.20	16.64 ± 5.29	0.222
CAT	6.87 ± 3.68	10.06 ± 3.42	**0.000**
Semantic fluency test	13.70 ± 8.48	22.12 ± 12.10	**0.000**
SAND naming	7.42 ± 4.31	10.32 ± 3.51	**0.003**
ROCF immediate copy	15.18 ± 9.48	21.05 ± 9.91	**0.007**
Phonemic fluency test	13.40 ± 10.75	22.18 ± 11.00	**0.000**
ADL	3.58 ± 2.21	4.73 ± 2.15	**0.011**
IADL	2.65 ± 2.24	4.61 ± 2.60	**0.000**

Statistically significant differences are indicated in bold

*ADL* activities of daily living, *BJLOT* Benton judgment of lines orientation task, *CAT* constructional apraxia task, *CINDD* Caregiver’s inventory neuropsychological diagnosis Dementia, *IADL* instrumental activities of daily living, *MMSE* mini mental state examination, *MOCA* montreal cognitive assessment, *NPI* neuropsychiatric inventory, *RAVLT* Rey’s auditory 15-word learning test, *ROCF* Rey–Osterrieth complex figure test, *SAND* screening for Aphasia in NeuroDegeneration

## Discussion

Herein, we propose the CINDD, a new tool directed to caregivers investigating ecologic cognitive and behavioral status of patients with cognitive impairment easily applicable in real-world clinical settings. The scale showed high acceptability since data were computable for the entire sample. The acceptability of the CINDD is also supported by the absence of both ceiling and floor effects. The internal consistency of the CINDD was shown to be high and acceptable (*α* = 0.969). All cognitive domains, the personality and social behavior domain, the ideation/perception domain, and the impact on activities of daily living domain presented high reliability. The low reliability of anxiety and mood domains may be due to the presence of the lowest number of items investigating these domains and suggests other scales are necessary to have sufficient information about these features. However, the presence of such two domains does not affect the overall reliability of the scale, which was indeed created as a screening tool. Moreover, the CINDD total score had moderate correlation with all items except with anxiety items.

As for convergent construct validity, we found a significant inverse correlation between the CINDD total score and both the MMSE and the MOCA the most frequently used cognitive screening tests. Furthermore, all the CINDD cognitive domains correlated with the corresponding cognitive tests except for the perceptive-spatial praxis skills domain not correlating with the BJLOT. This is probably due to the absence of specific questions investigating visuospatial skills in the CINDD. On the other hand, the CINDD perceptive-spatial praxis skills domain correlates with the cognitive test analyzing the visuocostructive abilities, such as CAT.

As for behavioral disturbances, we found that all behavioral CINDD domains correlated with the corresponding NPI items, except for apathy. We speculate that this is due to the complexity of the apathy construct needing more detailed scales to be assessed (Levyand Dubois 2006). We found also significant correlations between the CINDD impact on activities of daily living domain and traditional scores investigating functional autonomy suggested within the DSM-5 (APA 2013).

The compilation of CINDD did not appear influenced by age, education, resilience, depression, and quality of life of caregivers.

Finally, we looked for an indicative cut-off within the CINDD that can discriminate dementia from MCI, in order to better orient the clinician in planning the neuropsychological evaluation. By balancing sensitivity over specificity in line with the screening purpose of the scale, we found an indicative cut-off equal to 59 presented fair discriminatory power. The worst performances on a number of cognitive tests of patients with CINDD > 59 compared with patients with CINND < 59 strengthen our data. We failed to detect significant differences between such two groups for memory tests and for the BJLOT. As mentioned previously, the BJLOT may better evaluate visuospatial abilities which are not the main focus of the CINDD. As for memory tests, we speculate CINDD memory items; although able to capture memory dysfunction as shown by the good convergent construct validity, we cannot discriminate different severities of memory impairment, thus needing to be completed by patient-oriented cognitive tests. Administering the CINND to a group of healthy control is also warranted in order to understand the discriminatory power with MCI. However, this was out of the scope of the present preliminary study.

In summary, we here propose a new tool for screening cognitive and behavioral skills as well as interference on daily living easily applicable in real-life clinical settings. Such a tool is administered to caregivers and can be proposed to shorten the time of clinical neuropsychological interview and to quantify the collected information in both clinical and research settings. We demonstrated the CINDD is a reliable instrument to quantify the cognitive, functional, and behavioral difficulties of patients with cognitive impairment with fair discriminatory power between dementia and MCI. The latter information may be useful in planning the subsequent neuropsychological evaluations needed.

The assessment of cognitive functioning might also benefit from non-performance-based person-oriented assessments. Assessing cognitive functioning through interview-based methods is practical and might enable the examination of the impact of cognition on daily functioning. We believe associating the CINDD to formal cognitive testing may represent a fair compromise between the necessary data, the time consumed, and the patient's fatigue (Weintraub 2022).

### Supplementary Information

Below is the link to the electronic supplementary material.

Supplementary file1 (DOCX 36 KB)

Supplementary file2 (DOCX 41 KB)

Supplementary file3 (DOCX 13 KB)
