# Supplementary material for: Psychometric properties of the Caregiver’s inventory neuropsychological diagnosis dementia (CINDD) in mild cognitive impairment and dementia
Source: J Neural Transm (Vienna). 2024 Jan 10;131(2):173–80. doi: 10.1007/s00702-023-02728-0 (PMC10791830; doi:10.1007/s00702-023-02728-0)
Supplement: Supplementary file 1 — Supplementary file1 (DOCX 36 KB) [file 702_2023_2728_MOESM1_ESM.docx]

**CAREGIVER’S INVENTORY NEUROPSYCHOLOGICAL DIAGNOSIS DEMENTIA (CINDD)**

| Name and Surname ………………………………………………………………………………………  Education (years) ………………Sex M/F………………Age………….Exam Date………/………/….. |
| --- |

The clinician reports in the *Table_1* the total score for each sub-domain, obtained from the sum of the items, the score relating to the impact of specific deficits on daily living activities and the total CINDD score calculated by adding the 9 domains.

A summary *Table_2* has been inserted at the end of the scale, in which the examiner can note the altered domains, the order of appearance and the time interval elapsed between one or more domains of onset and subsequent ones. This Table_2 can help to build the peculiar (although not invariable) pattern of cognitive-behavioral domains with the temporal order of appearance, corresponding to a specific type of dementia.

***Table_1:* *TOTAL SCORE, SUB-DOMAINS AND IMPACT ON ACTIVITIES of DAILY LIVING***

| **Domains^§^** | **Subscores** | **“Impact on activities of daily living”**  **Subscores for single domain** |
| --- | --- | --- |
| ***1. Memory*** |  |  |
| ***2. Perceptual Spatial-Praxis Skills*** |  |  |
| ***3. Language*** |  |  |
| ***4. Executive Functions*** |  |  |
| ***5. Personality and social behaviour*** |  |  |
| ***6. Ideation/perception*** |  |  |
| ***7. Mood*** |  |  |
| ***8. Anxiety*** |  |  |
| ***9. Impact on activities of daily living_Total*** |  | ***--*** |
|  | | |
| ***TOTAL SCORE of CINDD: ____________________________________________________________________***  *(Calculate the sum of the 9 domains§)* | | |

**Blundo Carlo^1^, Ricci Monica^1^**

^1^Department of Neuroscience, Center of Cognitive Disorders and Dementia, San Camillo Hospital, Rome, Italy

**Compilation instructions:** Mark with a cross the cell corresponding to the score related to the symptom severity

**0** = non present **1**= minor or rarely **2**= moderate or occasional **3**= severe or frequently

Questions should refer to changes that occurred after the onset of the illness concerning the last 30-60 days.

| **MEMORY** | | | | |
| --- | --- | --- | --- | --- |
| **1.Difficulty remembering recent events**  The patient shows that they do not remember episodes that have occurred in the last week, cannot say what they have eaten, who they have met, who they spoke to on the phone one, two days previously | 0 | 1 | 2 | 3 |
| **2. Difficulty remembering and learning new information**  The patient shows that they do not remember things read or seen on TV, cannot mentally retain a shopping list | 0 | 1 | 2 | 3 |
| **3. Repetitive behaviour and speech**  The patient returns to topics already discussed or repeats things previously mentioned shortly before | 0 | 1 | 2 | 3 |
| **4. Difficulty remembering the place of objects**  The patient forgets where they placed or stored objects shortly before and spends a lot of time finding them | 0 | 1 | 2 | 3 |
| **5. Difficulty remembering appointments and deadlines**  The patient is unable to remember important dates of payments or events, or the time and address of the same | 0 | 1 | 2 | 3 |
| **HOW MUCH DOES MEMORY DISORDER AFFECT ACTIVITIES OF DAILY LIVING?**  Global assessment. This Item considers the overall severity of symptoms related to this area. | 0 | 1 | 2 | 3 |

| **PERCEPTUAL-SPATIAL AND PRAXIS SKILLS** | | | | |
| --- | --- | --- | --- | --- |
| **1.Difficulty orienting in unknown places**  The patient has trouble orienting themselves in places they do not know or rarely visit, e.g., in a strange neighbourhood | 0 | 1 | 2 | 3 |
| **2.Difficulty in orientation in familiar places outside patient’s home**  The patient shows a lack of recognition and tends to get lost in their own neighbourhood or in well-known places | 0 | 1 | 2 | 3 |
| **3.Difficulty in orientation in patient’s home**  The patient shows that they do not recognize the different rooms in their own home, e.g., they confuse the bedroom with the bathroom, etc. | 0 | 1 | 2 | 3 |
| **4.Difficulty recognizing faces of familiar people or the architecture, structure of familiar buildings**  The patient is unable to recognize faces of family members, close friends, does not recognize places they already know. | 0 | 1 | 2 | 3 |
| **5.Difficulty recognizing coins or banknotes**  The patient is unable to handle money, makes mistakes in payments interchanges banknotes or attributes the wrong value to them. | 0 | 1 | 2 | 3 |
| **6.Difficulty in dressing, making the bed, or setting the table**  The patient mistakes the sequence in putting on clothing, mistakes the order of bed sheets and blankets, and fails to put the tablecloth on the table in the right way. | 0 | 1 | 2 | 3 |
| **7.Inaccuracy in reaching for objects with the hand.**  The patient "fumbles" when pouring liquid, also use when using cutlery, etc. | 0 | 1 | 2 | 3 |
| **8. Difficulty in writing and reading**  The patient finds difficulty in writing aligned, misses the point when reading, has trouble finding the line where they were reading, gets figures wrong in columns when they have to do a calculation. | 0 | 1 | 2 | 3 |
| **9. Praxic difficulties**  The patient has difficulty using utensils (e.g. kitchen or work tools), has difficulty writing, is no longer able to make complex gestures (e.g., the sign of the cross) | 0 | 1 | 2 | 3 |
| **10. Difficulty locating objects in space**  The patient has difficulty locating objects placed in front of them, e.g. in finding immediately a food item in the refrigerator finding a specific product among similar products in the supermarket. | 0 | 1 | 2 | 3 |
| **HOW MUCH DOES THE DISORDER INTERFERE WITH ACTIVITIES OF DAILY LIVING?**  Global Assessment. This item considers the overall severity of symptoms related to this area. | 0 | 1 | 2 | 3 |

VVVV

| **EXECUTIVE FUNCTIONS** | | | | |
| --- | --- | --- | --- | --- |
| **1.Difficulty finding words during speech**  While speaking the patient cannot find specific words, resorts to turns of phrase, often says "thing" | 0 | 1 | 2 | 3 |
| **2.Reduced verbal production**  Patient speaks less, sentences are short, often not finished, speech is characterized  by an  increase in pauses | 0 | 1 | 2 | 3 |
| **3.Ungrammatical speech (agrammatism)**  The patient makes grammatical errors, tends to speak like a non-native language speaker | 0 | 1 | 2 | 3 |
| **4.Difficulty articulating words, hesitation in speaking, stuttering (verbal apraxia)**  Patient speaks in a laboured and slow manner, slurs and repeats a phoneme several times before pronouncing the exact word e.g. to say street, initially says "stre..." then "stri..." and finally "street" | 0 | 1 | 2 | 3 |
| **5.Language fluent but poor in information content (Phonological-Semantic Deficit)**  The patient speaks much as before but the speech content is poor, poorly informative or even poorly understandable. Gets words wrong, e.g., says apple instead of pear or instead of chair or "tible” instead of table, says words that do not exist in the English language | 0 | 1 | 2 | 3 |
| **6.Deficits in understanding word meanings and object recognition**  The patient does not know the meaning of many words, has difficulty naming common objects, does not recognize them and cannot indicate what they are used for | 0 | 1 | 2 | 3 |
| **7.Difficulty in reading and writing**  The patient has difficulty reading and/or writing despite being able to write aligned, and to not lose their place when reading | 0 | 1 | 2 | 3 |
| **8.Difficulty performing calculations**  The patient is unable to perform addition, subtraction, multiplication, and division to one or two digits | 0 | 1 | 2 | 3 |
| **HOW MUCH DOES THE LANGUAGE DISORDER INTERFERE WITH DAILY LIVING?**  Global Assessment. This item considers the overall severity of symptoms related to this area | 0 | 1 | 2 | 3 |

VVVV

| **PERSONALITY AND SOCIAL BEHAVIOUR** | | | | |
| --- | --- | --- | --- | --- |
| **1.Aggression is present**  The patient shows verbal and/or physical aggression, shouts in an angry tone, threatens or raises hands, throws objects, kicks or punches furniture | 0 | 1 | 2 | 3 |
| **2.Hyperorality is present**  In terms of daily frequency and amount the patient presents increased consumption of food and drinks particularly sweet food, of smoking, alcohol, also frequently hold objects of various types in their mouth | 0 | 1 | 2 | 3 |
| **3.Dependence on the environment is present**  The patient has a need to touch, to use, objects, tools that they see in front of them without reason or without invitation or to imitate the behaviour of a person close to them | 0 | 1 | 2 | 3 |
| **4.Irritability is present**  The patient loses their temper over trivial matters, gets angry easily, gets nervous if they have to queue or wait for someone | 0 | 1 | 2 | 3 |
| **5.Inappropriate joking is present**  Patient makes inappropriate or offensive jokes, often plays tricks or jokes for no reason | 0 | 1 | 2 | 3 |
| **6.Impulsiveness is present**  The patient speaks or acts without considering the consequences of their actions or words | 0 | 1 | 2 | 3 |
| **7.Disinhibition is present**  The patient appears less reserved, speaks easily with strangers as if they know them, reveals private facts in public, says things that are embarrassing to others or even offensive, is less respectful of social rules | 0 | 1 | 2 | 3 |
| **8.Motor restlessness is present**  The patient cannot keep quiet, tends to continuously engage in chores without any real need, e.g. walks up and down without a purpose, cannot sit still, wanders around the house for no reason. | 0 | 1 | 2 | 3 |
| **9.Tendency to wander is present.**  The patient tends to move around without a definite purpose and goal, both in and out of the house. | 0 | 1 | 2 | 3 |
| **10.Neglect of personal care is present**  The patient does not engage in personal cleanliness, dresses poorly, and neglects clothing. | 0 | 1 | 2 | 3 |
| **11.Apathy is present**  The patient shows no interest in household chores, in regard to visits from friends, his or her hobbies or previous interests, tends to be passive to the point of not reacting with respect to any stimulus from the surrounding environment. This condition should not be associated with sadness, crying, depression. | 0 | 1 | 2 | 3 |
| **12.Anergy is present**  The patient tends to sit still even for hours without spontaneously initiating any kind of activity. | 0 | 1 | 2 | 3 |
| **13.Inadequate emotional response is present.**  The patient demonstrates indifference to both sad and happy events in their past or present | 0 | 1 | 2 | 3 |
| **14.Incontinence is present**  The patient experiences urine leaks without realizing it. The disorder does not depend on disease of the bladder. | 0 | 1 | 2 | 3 |
| **HOW MUCH DO PERSONALITY AND SOCIAL BEHAVIOUR DISORDERS**  **INTERFERE WITH ACTIVITIES OF DAILY LIVING?**  Global assessment. This item considers the overall severity of symptoms related to this area. | 0 | 1 | 2 | 3 |

x

| **IDEATION/PERCEPTION** | | | | |
| --- | --- | --- | --- | --- |
| **1.Suspiciousness is present**  Patient shows distrust, tends to make repeated checks even with respect to family members | 0 | 1 | 2 | 3 |
| **2.Delusions of persecution are present**  The patient is genuinely convinced that they are in a dangerous situation, that someone wants to harm them, plots behind their back. | 0 | 1 | 2 | 3 |
| **3.Delusions of theft are present**  The patient is genuinely convinced (not just suspiciousness) that someone is stealing from them, accuses family members of stealing their belongings, is inclined to put everything under lock and key | 0 | 1 | 2 | 3 |
| **4.Visual hallucinations are present**  The patient says they see things that others do not see, e.g., absent family members, animals, people, strangers in the house. | 0 | 1 | 2 | 3 |
| **5.Auditory hallucinations are present**  The patient says they hear voices that others do not, sometimes does things that the "voices ask me to do," talks to people who are not present. | 0 | 1 | 2 | 3 |
| **6.False recognition is present**  The patient does not recognize a family member and believes they are an impostor or look-alike, believes events observed on TV really happen, is convinced that the place where they live is not their own home | 0 | 1 | 2 | 3 |
| **HOW MUCH DO IDEATION/PERCEPTION DISORDERS INTERFERE WITH ACTIVITIES OF DAILY LIVING?**  Overall rating. This item considers the overall severity of symptoms related to this area. | 0 | 1 | 2 | 3 |

x

| **MOOD** | | | | |
| --- | --- | --- | --- | --- |
| **1.A reduction in mood is present.**  The patient appears sad, hopeless for the future, feels like a failure, cries | 0 | 1 | 2 | 3 |
| **2.An increase in mood is present.**  The patient appears too happy, is always cheerful, euphoric for no reason | 0 | 1 | 2 | 3 |
| **HOW MUCH DOES THE MOOD DISORDER INTERFERE WITH ACTIVITIES OF DAILY LIVING?**  Global assessment. This item considers the overall severity of symptoms related to this area | 0 | 1 | 2 | 3 |

x

| **ANXIETY** | | | | |
| --- | --- | --- | --- | --- |
| **1.Anxiety is present**  The patient is unable to relax, is always tense, is often afraid that something bad will happen to themselves or their family | 0 | 1 | 2 | 3 |
| **2.Phobias are present**  The patient has begun to have unfounded fears (e.g., fear of traveling, of being alone, of being in crowds or enclosed places) | 0 | 1 | 2 | 3 |
| **HOW MUCH DOES THE ANXIETY DISORDER INTERFERE WITH ACTIVITIES OF DAILY LIVING?**  Global assessment. This item considers the overall severity of symptoms related to this area | 0 | 1 | 2 | 3 |

**x**

| **RELIABILITY**  **Instructions for compilation:**  indicates the source of origin of the information regarding the patient's clinical status and its degree of reliability. | |
| --- | --- |
| **Interview sources:**   - Family - Friends - Other | **Reliability:**   1. = low 2. = just enough 3. = sufficient 4. = good 5. = very good |

**Table_2: SUMMARY PROFILE WITH ORDER OF APPEARANCE OF THE SYMPTOMS IN DEMENTIA ACCORDING TO THE “CINDD”**

| **Instructions for compilation:**  Mark the presence [YES] or absence [NO] of one or more pathological domains at the onset of dementia and the total score. Repeat the procedure with the pathological domain(s) that appeared after the initial symptom(s). Mark the time elapsed between the appearance of the initial symptom(s) and the subsequent symptom(s). | | | | | | | | | | |
| --- | --- | --- | --- | --- | --- | --- | --- | --- | --- | --- |
| **DOMAINS** | **Onset**  **Symptom (s)** | | **Score** | **Time since**  **first symptom(s)**  *(in months)* | | | | **Next**  **Symptom (s)** | | **Score** |
| **MEMORY** | **YES** | **NO** |  | 0-6 | 7-12 | 13-24 | >24 | **YES** | **NO** |  |
| **PERCEPTUAL SPATIAL PRAXIS SKILLS** | **YES** | **NO** |  | 0-6 | 7-12 | 13-24 | >24 | **YES** | **NO** |  |
| **LANGUAGE** | **YES** | **NO** |  | 0-6 | 7-12 | 13-24 | >24 | **YES** | **NO** |  |
| **EXECUTIVE FUNCTIONS** | **YES** | **NO** |  | 0-6 | 7-12 | 13-24 | >24 | **YES** | **NO** |  |
| **PERSONALITY/SOCIAL BEHAVIOUR** | **YES** | **NO** |  | 0-6 | 7-12 | 13-24 | >24 | **YES** | **NO** |  |
| **IDEATION/**  **PERCEPTION** | **YES** | **NO** |  | 0-6 | 7-12 | 13-24 | >24 | **YES** | **NO** |  |
| **MOOD** | **YES** | **NO** |  | 0-6 | 7-12 | 13-24 | >24 | **YES** | **NO** |  |
| **ANXIETY** | **YES** | **NO** |  | 0-6 | 7-12 | 13-24 | >24 | **YES** | **NO** |  |
