# Supplementary material for: Psychometric properties of the Caregiver’s inventory neuropsychological diagnosis dementia (CINDD) in mild cognitive impairment and dementia
Source: J Neural Transm (Vienna). 2024 Jan 10;131(2):173–80. doi: 10.1007/s00702-023-02728-0 (PMC10791830; doi:10.1007/s00702-023-02728-0)
Supplement: Supplementary file 2 — Supplementary file2 (DOCX 41 KB) [file 702_2023_2728_MOESM2_ESM.docx]

**CAREGIVER’S INVENTORY NEUROPSYCHOLOGICAL DIAGNOSIS DEMENTIA (CINDD)**

Nome e Cognome .………………………………………………………………………………………………………………………..

Scolarità (anni) ….…………………………………………… Sesso M/F……………… Età……………………

Data dell’esame ……./………../………..

Il clinico riporta in *Tabella_1* il punteggio totale per singolo sotto-dominio, ottenuto dalla somma degli item, il punteggio relativo all’impatto dei deficit specifici sulle attività di vita quotidiana ed il punteggio totale della CINDD calcolato facendo la somma dei 9 domini.

Alla fine della scala è stata inserita la *Tabella_2* riassuntiva, in cui l’esaminatore può annotare i domini alterati, l’ordine di comparsa e l’intervallo di tempo trascorso tra uno o più domini di esordio e quelli successivi. Tale Tabella_2 può aiutare a costruire il peculiare (anche se non invariabile) pattern dei domini cognitivo-comportamentali con l’ordine temporale di comparsa, corrispondente a un determinato tipo di demenza.

***Tabella_1: PUNTEGGIO TOTALE, SOTTO-DOMINI E IMPATTO SULLE ATTIVITÁ DI VITA QUOTIDIANA***

| **Dominio^§^** | **Punteggio**  **sotto-domini** | **Punteggio item**  **“Impatto sulle attività di vita quotidiana” per singolo dominio** |
| --- | --- | --- |
| ***1. Memoria*** |  |  |
| ***2. Abilità percettive spaziali e prassiche*** |  |  |
| ***3. Linguaggio*** |  |  |
| ***4. Funzioni esecutive*** |  |  |
| ***5. Personalità e comportamento sociale*** |  |  |
| ***6. Ideazione/Percezione*** |  |  |
| ***7. Umore*** |  |  |
| ***8. Ansia*** |  |  |
| ***9. Impatto sulle attività di vita quotidiana: totale*** |  | ***---*** |
| ***Punteggio totale della CINDD: ________________________________________________________***  *(Calcola la somma dei 9 domini^§^)* | | |

**Blundo Carlo^1^, Ricci Monica^1^**

^1^Department of Neuroscience, Center of Cognitive Disorders and Dementia, San Camillo Hospital, Rome, Italy

**Istruzioni per la compilazione**:

segnare con una crocetta la cella corrispondente al punteggio relativo alla gravità del sintomo.

**0**= Assente  **1**=Lieve o raramente  **2**=Moderato o occasionale **3**=Grave o frequente

Le domande devono fare riferimento a modificazioni insorte dopo l’inizio della malattia riguardante gli ultimi 30-60 giorni.

| **MEMORIA** | | | | |
| --- | --- | --- | --- | --- |
| **1. Difficoltà nel ricordare avvenimenti recenti**  Il paziente mostra di non ricordare episodi accaduti nell’ultima settimana, non sa dire cosa ha mangiato, chi ha incontrato, chi gli/le ha parlato a telefono uno, due giorni prima. | 0 | 1 | 2 | 3 |
| **2. Difficoltà nel ricordare e apprendere nuove informazioni**  Il paziente mostra di non ricordare le cose lette o viste in TV, di tenere a mente la lista della spesa. | 0 | 1 | 2 | 3 |
| **3. Comportamento ed eloquio ripetitivo**  Il paziente ritorna su argomenti già discussi o richiede cose dette poco prima. | 0 | 1 | 2 | 3 |
| **4. Difficoltà nel ricordare dove ha posto gli oggetti**  Il paziente dimentica dove, poco prima, ha poggiato o conservato oggetti e trascorre molto tempo per trovarli. | 0 | 1 | 2 | 3 |
| **5. Difficoltà nel ricordare appuntamenti e scadenze**  Il paziente è incapace di tenere a mente date importanti di pagamenti o ricorrenze, l’orario e l’indirizzo degli stessi. | 0 | 1 | 2 | 3 |
| **QUANTO IL DISTURBO DI MEMORIA INTERFERISCE SULLE ATTIVITÀ DELLA VITA QUOTIDIANA?**  Valutazione globale. Questo Item considera la gravità complessiva dei sintomi relativi a quest’area. | 0 | 1 | 2 | 3 |

| **ABILITA’ PERCETTIVE- SPAZIALI E PRASSICHE** | | | | |
| --- | --- | --- | --- | --- |
| **1. Difficoltà di orientamento in luoghi sconosciuti**  Il paziente ha problemi ad orientarsi in luoghi che non conosce o che frequenta di rado, ad es. in un quartiere non suo. | 0 | 1 | 2 | 3 |
| **2. Difficoltà di orientamento in luoghi familiari al di fuori della propria abitazione**  Il paziente mostra di non riconoscere e tende a perdersi nel suo quartiere o in luoghi ben conosciuti. | 0 | 1 | 2 | 3 |
| **3. Difficoltà di orientamento nella propria abitazione**  Il paziente mostra di non riconoscere i differenti ambienti della propria casa, ad es. confonde la camera da letto con il bagno ecc. | 0 | 1 | 2 | 3 |
| **4. Difficoltà a riconoscere volti di persone familiari o l’architettura, struttura di edifici conosciuti**  Il paziente è incapace di riconoscere facce di familiari, di amici intimi, non riconosce luoghi a lui noti. | 0 | 1 | 2 | 3 |
| **5. Difficoltà nel riconoscere monete o banconote**  Il paziente non è in grado di gestire il denaro, commette errori nei pagamenti scambia le banconote o attribuisce  ad esse un valore sbagliato. | 0 | 1 | 2 | 3 |
| **6. Difficoltà nel vestirsi, rifare il letto, o nell’apparecchiare la tavola**  Il paziente sbaglia la sequenza nell’indossare i capi di abbigliamento, sbaglia la sistemazione delle lenzuola, delle coperte e non riesce a disporre sulla tavola la tovaglia nel verso giusto. | 0 | 1 | 2 | 3 |
| **7.Imprecisione nel raggiungere con la mano gli oggetti**  Il paziente “va a tentoni” per versare un liquido, per utilizzare la posata nel piatto ecc. | 0 | 1 | 2 | 3 |
| **8. Difficoltà nella scrittura e nella lettura**  Il paziente trova difficoltà nello scrivere allineato, quando legge tende a perdere il segno, stenta a trovare la riga dove stava leggendo, si sbaglia a mettere le cifre in colonna quando deve fare un calcolo. | 0 | 1 | 2 | 3 |
| **9. Difficoltà prassiche**  Il paziente ha difficoltà ad usare utensili (ad es.di cucina o strumenti di lavoro), ha difficoltà di scrittura, non è più in grado di compiere movimenti complessi (ad esempio il segno della croce) | 0 | 1 | 2 | 3 |
| **10. Difficoltà di localizzazione di oggetti nello spazio**  Il paziente ha difficoltà a localizzare oggetti posti di fronte a lui, ad es. nel trovare subito un alimento nel  frigorifero trovare uno specifico prodotto tra prodotti simili al supermercato. | 0 | 1 | 2 | 3 |
| **QUANTO IL DISTURBO INTERFERISCE SULLE ATTIVITÀ DELLA VITA QUOTIDIANA?**  Valutazione globale. Questo Item considera la gravità complessiva dei sintomi relativi a quest’area. | 0 | 1 | 2 | 3 |

| **LINGUAGGIO** | | | | |
| --- | --- | --- | --- | --- |
| **1. Difficoltà a trovare le parole durante un discorso (anomie)**  Il paziente mentre parla non trova le parole specifiche, per questo ricorre a giri di parole, dice spesso  ”coso/a” | 0 | 1 | 2 | 3 |
| **2. Produzione verbale ridotta (logopenia)**  Il paziente parla meno, le frasi sono brevi, spesso non vengono terminate, l’eloquio è caratterizzato  da un aumento delle pause | 0 | 1 | 2 | 3 |
| **3. linguaggio sgrammaticato (agrammatismo)**  Il paziente nell’esprimersi commette errori di grammatica, tende a parlare come uno straniero | 0 | 1 | 2 | 3 |
| **4. Difficoltà ad articolare le parole, esitazione nel parlare, è presente balbettio (aprassia verbale)**  Il paziente parla in modo difficoltoso e rallentato, tartaglia e ripete più volte un fonema prima di pronunciare con esattezza la parola ad es. per dire strada prima dice “stre…” poi “stri…” ed infine  “strada” | 0 | 1 | 2 | 3 |
| **5. linguaggio fluente ma povero nel contenuto informativo (Deficit fonologico-semantico)**  Il paziente parla come o più di prima ma il contenuto del discorso è povero, scarsamente informativo o addirittura poco comprensibile. Sbaglia le parole, ad es., dice mela invece di pera o invece di sedia o “tivolo” al posto di tavolo, dice parole inesistenti nella lingua italiana | 0 | 1 | 2 | 3 |
| **6. Deficit di comprensione di significati di parole e di riconoscimento di oggetti**  Il paziente non sa il significato di molte parole, ha difficoltà a denominare oggetti comuni, non li riconosce e non sa indicare a cosa servono | 0 | 1 | 2 | 3 |
| **7. Difficoltà a leggere e scrivere**  Il paziente ha difficoltà a leggere e/o scrivere pur essendo in grado di scrivere allineato e di non perdere la riga quando legge | 0 | 1 | 2 | 3 |
| **8. Difficoltà nello svolgere calcoli**  Il paziente non riesce ad eseguire addizioni, sottrazioni, moltiplicazioni e divisioni ad una cifra o due  Cifre | 0 | 1 | 2 | 3 |
| **QUANTO IL DISTURBO DEL LINGUAGGIO INTERFERISCE SULLA VITA QUOTIDIANA?**  Valutazione globale. Questo item considera la gravità complessiva dei sintomi relativi a questa area | 0 | 1 | 2 | 3 |

| **ABILITA’ ESECUTIVE** | | | | |
| --- | --- | --- | --- | --- |
| **1. E’ presente incapacità nel pianificare e programmare le comuni attività**  Il paziente non riesce a portare a termine programmi di lavoro o discorsi, risulta inconcludente, non è in grado di costruire una sequenza di azioni secondo un criterio funzionale per raggiungere uno scopo | 0 | 1 | 2 | 3 |
| **2. E’ presente disattenzione**  Il paziente non riesce a concentrarsi su una sola attività (ad es. leggere, guardare la TV, fare un discorso, lavorare), si distrae, perde il filo | 0 | 1 | 2 | 3 |
| **3. E’ presente inflessibilità**  Il paziente con difficoltà cambia opinione, è cocciuto, non cambia modi di pensare ed agire per far fronte alle situazioni | 0 | 1 | 2 | 3 |
| **4. E’ presente mancanza di insight**  Il paziente non è consapevole dei suoi deficit prestazionali, non ritiene di essere malato | 0 | 1 | 2 | 3 |
| **5. E’ presente perseverazione**  Il paziente è divenuto ripetitivo, ripete gli stessi discorsi o fa insistentemente le stesse domande, fa in continuazione le stesse cose (ad es. va più volte in bagno, si abbottona e sbottona, si veste e si sveste in continuazione, manipola oggetti secondo una stessa sequenza più volte, attorciglia carta, nastri | 0 | 1 | 2 | 3 |
| **6. E’ presente mancanza di critica e di giudizio**  Il paziente non è consapevole di commettere errori o di riconoscere sbagli nel comportamento degli altri, fa delle scelte e prende delle decisioni in modo avventato | 0 | 1 | 2 | 3 |
| **QUANTO IL DISTURBO DELLE FUNZIONI ESECUTIVE INTERFERISCE SULLA VITA QUOTIDIANA?**  Valutazione globale. Questo item considera la gravità complessiva dei sintomi relativi a questa area | 0 | 1 | 2 | 3 |

| **PERSONALITA’ E COMPORTAMENTO SOCIALE** | | | | |
| --- | --- | --- | --- | --- |
| **1. E’ presente aggressività**  Il paziente mostra aggressività verbale e/o fisica, grida con tono arrabbiato, minaccia o alza le mani, scaglia oggetti, prende a calci o a pugni i mobili | 0 | 1 | 2 | 3 |
| **2. E’ presente iperoralità**  Il paziente presenta un aumento, per frequenza giornaliera e per quantità, dell’assunzione di cibo e bevande, soprattutto alimenti dolci, di fumo, di alcool, inoltre porta spesso alla bocca oggetti di vario tipo | 0 | 1 | 2 | 3 |
| **3. E’ presente dipendenza dall’ambiente**  Il paziente ha bisogno di toccare, di usare senza ragione di farlo o senza invito, oggetti, strumenti che vede davanti a sé oppure di imitare i comportamenti di una persona vicino a sé | 0 | 1 | 2 | 3 |
| **4. E’ presente irritabilità**  Il paziente perde la calma per questioni di poco conto, si arrabbia facilmente, si innervosisce se deve fare una fila, attendere qualcuno | 0 | 1 | 2 | 3 |
| **5. E’ presente una scherzosità inadeguata**  Il paziente fa battute inadeguate o offensive, spesso gioca o scherza senza motivo | 0 | 1 | 2 | 3 |
| **6. E’ presente impulsività**  Il paziente parla o agisce senza considerare le conseguenze delle sue azioni o parole | 0 | 1 | 2 | 3 |
| **7. E’ presente disinibizione**  Il paziente appare meno riservato, parla facilmente con estranei come se li conoscesse, racconta in pubblico fatti privati, dice cose che creano imbarazzo negli altri o addirittura offensive, è meno rispettoso delle regole sociali | 0 | 1 | 2 | 3 |
| **8. E’ presente irrequietezza motoria**  Il paziente non riesce a stare tranquillo, tende ad impegnarsi di continuo in faccende senza che esista una reale necessità, ad es. cammina su e giù senza uno scopo, non riesce a stare seduto, va frugando qua e là in casa senza motivo. | 0 | 1 | 2 | 3 |
| **9. E’ presente la tendenza al vagabondaggio**  Il paziente tende a muoversi senza uno scopo ed una meta precisa, sia dentro che fuori casa. | 0 | 1 | 2 | 3 |
| **10. E’ presente trascuratezza nella cura della persona**  Il paziente non si dedica alla pulizia personale, si veste male, ha un abbigliamento trascurato. | 0 | 1 | 2 | 3 |
| **11. E’ presente apatia**  Il paziente non mostra interesse verso le faccende di casa, nei riguardi di visite di amici, dei suoi hobby o di interessi precedenti, tende ad essere passivo fino a non reagire rispetto a qualsiasi  stimolo dell’ambiente circostante. Questa condizione non deve associarsi a tristezza, pianto, depressione. | 0 | 1 | 2 | 3 |
| **12. E’ presente anergia**  Il paziente tende a restare fermo anche per ore senza iniziare spontaneamente nessun tipo di attività. | 0 | 1 | 2 | 3 |
| **13. E’ presente una risposta emotiva inadeguata**  Il paziente dimostra indifferenza rispetto agli eventi sia tristi che felici della sua vita trascorsa ed attuale | 0 | 1 | 2 | 3 |
| **14. E’ presente incontinenza**  Il paziente tende a perdere le urine senza rendersene conto. Il disturbo non dipende da malattia della vescica. | 0 | 1 | 2 | 3 |
| **QUANTO I DISTURBI DELLA PERSONALITA’ E DEL COMPORTAMENTO SOCIALE INTERFERISCONO SULLE ATTIVITA’ DELLA VITA QUOTIDIANA?**  Valutazione globale. Questo item considera la gravità complessiva dei sintomi relativi a questa area. | 0 | 1 | 2 | 3 |

| **IDEAZIONE / PERCEZIONE** | | | | |
| --- | --- | --- | --- | --- |
| **1. E’ presente sospettosità**  Il paziente mostra di non fidarsi, tende a fare ripetuti controlli anche rispetto a familiari | 0 | 1 | 2 | 3 |
| **2. Sono presenti deliri di persecuzione**  Il paziente è realmente convinto di stare in una situazione di pericolo, che qualcuno gli voglia fare del male, complotti alle sue spalle. | 0 | 1 | 2 | 3 |
| **3. Sono presenti deliri di furto**  Il paziente è realmente convinto (non si tratta solo di sospettosità) che qualcuno lo stia derubando,  accusa i familiari di furti dei suoi oggetti, è portato a mettere ogni cosa sottochiave | 0 | 1 | 2 | 3 |
| **4. Sono presenti allucinazioni visive**  Il paziente dice di vedere cose che gli altri non vedono, ad es. familiari assenti, animali, persone estranee in casa. | 0 | 1 | 2 | 3 |
| **5. Sono presenti allucinazioni uditive**  Il paziente dice di sentire voci che gli altri non sentono, talvolta fa cose che le “voci chiedono di fare”, parla con persone che non sono presenti. | 0 | 1 | 2 | 3 |
| **6. Sono presenti falsi riconoscimenti**  Il paziente non riconosce un familiare e crede che sia un impostore o un sosia, crede che gli eventi osservati in TV accadano realmente, è convinto che il posto in cui vive non sia la propria casa | 0 | 1 | 2 | 2 |
| **QUANTO I DISTURBI DELL’IDEAZIONE/PERCEZIONE INTERFERISCONO SULLE ATTIVITA’ DELLA VITA QUOTIDIANA?**  Valutazione globale. Questo item considera la gravità complessiva dei sintomi relativi a questa area. | 0 | 1 | 2 | 3 |

| **UMORE** | | | | |
| --- | --- | --- | --- | --- |
| **1. E’ presente una riduzione dell’umore**  Il paziente appare triste, senza speranza per il futuro, si sente un fallito, piange | 0 | 1 | 2 | 3 |
| **2. E’ presente un aumento del tono dell’umore**  Il paziente appare troppo felice, è sempre allegro, euforico senza motivo | 0 | 1 | 2 | 3 |
| **QUANTO IL DISTURBO DELL’UMORE INTERFERISCE SULLE ATTIVITA’ DELLA VITA QUOTIDIANA?**  Valutazione globale. Questo item considera la gravità complessiva dei sintomi relativi a questa area | 0 | 1 | 2 | 3 |

| **ANSIA** | | | | |
| --- | --- | --- | --- | --- |
| **1. E’ presente ansia**  Il paziente non è capace a rilassarsi, è sempre in tensione, ha spesso paura che possa accadere qualcosa di brutto a sé o ai familiari | 0 | 1 | 2 | 3 |
| **2. Sono presenti fobie**  Il paziente ha cominciato ad avere paure immotivate (ad es. paura di viaggiare, di restare solo, di stare tra la folla o in luoghi chiusi) | 0 | 1 | 2 | 3 |
| **QUANTO IL DISTURBO D’ANSIA INTERFERISCE SULLE ATTIVITA’ DELLA VITA QUOTIDIANA?**  Valutazione globale. Questo item considera la gravità complessiva dei sintomi relativi a questa area. | 0 | 1 | 2 | 3 |

| **ATTENDIBILITÁ**  **Istruzioni per la compilazione:**  indicare la fonte di provenienza delle informazioni riguardanti lo stato clinico del paziente e il suo grado di attendibilità. | |
| --- | --- |
| **Fonte dell’intervista:**   - famiglia - amici - altro | **Attendibilità della fonte:**   1. = bassa 2. = appena sufficiente 3. = sufficiente 4. = buona 5. = molto buona |

**Tabella_2: PROFILO RIASSUNTIVO CON ORDINE DI COMPARSA DEI SINTOMI NELLA DEMENZA SECONDO LA “CINDD”**

| **Istruzioni per la compilazione:**  Contrassegnare la presenza [SI] o l’assenza [NO] di uno o più domini patologici all’esordio della demenza  insieme al punteggio totale. Ripetere la procedura con il/i dominio/i patologici comparsi successivamente  al/ai sintomi di esordio. Contrassegnare il tempo trascorso tra la comparsa del/i sintomo/i d’esordio ed il/i successivo/i. | | | | | | | | | | |
| --- | --- | --- | --- | --- | --- | --- | --- | --- | --- | --- |
| **DOMINIO** | **Sintomo/i**  **di esordio** | | **Punteggio** | **Tempo trascorso**  **dal/i primo/i sintomo/i**  *(in mesi)* | | | | **Sintomo/i**  **Successivo/i** | | **Punteggio** |
| **MEMORIA** | **SI** | **NO** |  | 0-6 | 7-12 | 13-24 | >24 | **SI** | **NO** |  |
| **ABILITÁ PERCETTIVE SPAZILI E PRASSICHE** | **SI** | **NO** |  | 0-6 | 7-12 | 13-24 | >24 | **SI** | **NO** |  |
| **LINGUAGGIO** | **SI** | **NO** |  | 0-6 | 7-12 | 13-24 | >24 | **SI** | **NO** |  |
| **ABILITÁ ESECUTIVE** | **SI** | **NO** |  | 0-6 | 7-12 | 13-24 | >24 | **SI** | **NO** |  |
| **PERSONALITÁ/COMPORTAMENTO SOCIALE** | **SI** | **NO** |  | 0-6 | 7-12 | 13-24 | >24 | **SI** | **NO** |  |
| **IDEAZIONE/**  **PERCEZIONE** | **SI** | **NO** |  | 0-6 | 7-12 | 13-24 | >24 | **SI** | **NO** |  |
| **UMORE** | **SI** | **NO** |  | 0-6 | 7-12 | 13-24 | >24 | **SI** | **NO** |  |
| **ANSIA** | **SI** | **NO** |  | 0-6 | 7-12 | 13-24 | >24 | **SI** | **NO** |  |
