# Supplementary material for: Psychometric properties of the Caregiver’s inventory neuropsychological diagnosis dementia (CINDD) in mild cognitive impairment and dementia
Source: J Neural Transm (Vienna). 2024 Jan 10;131(2):173–80. doi: 10.1007/s00702-023-02728-0 (PMC10791830; doi:10.1007/s00702-023-02728-0)
Supplement: Supplementary file 3 — Supplementary file3 (DOCX 13 KB) [file 702_2023_2728_MOESM3_ESM.docx]

**Appendix_2**

**Demographic and clinical characteristics of caregivers divided accordint to patients’s diagnosis (D vs MCI), measured by T-test and Chi^2^ test.**

|  | **D**  mean±SD | **MCI**  mean±SD | **p** |
| --- | --- | --- | --- |
| **Age_cr** | 53.88±15.73 | 47.93±10.94 | 0.19 |
| **Education_cr** | 12.11±6.01 | 14.37±5.07 | 0.21 |
| **RS-14_cr** | 80.51±17.41 | 83.87±8.79 | 0.47 |
| **EQ-5D_cr** | 999.74±0.38 | 999.87±0.19 | 0.24 |
| **VAS_cr** | 75.88±19.51 | 79.25±18.28 | 0.57 |
| **BDI-II_cr** | 7.48±6.93 | 5.25±6.08 | 0.29 |
|  | | | |
| **Sex (M%)** | 25.9% | 31.3% | 0.71 |

**Abbreviations:** BDI-II, Beck Depression Inventory Second Edition; cr, caregivers; D, dementia; EQ-5D, EuroQol- 5 Dimension (EQ-5D); M, males; MCI, mild cognitive impairment; p, p-value; RS-14, 14-item Resilience Scale; SD, standard deviation; VAS, Visual Analog Scale.

**Pearson’s correlations among demographic and clinical characteristics of caregivers and CINDD total score.**

|  | **CINDD total score** | |
| --- | --- | --- |
|  | **r** | **p** |
| **Age_cr** | 0.104 | 0.51 |
| **Education_cr** | -0.027 | 0.87 |
| **RS-14_cr** | 0.004 | 0.98 |
| **EQ-5D_cr** | -0.151 | 0.34 |
| **VAS_cr** | -0.055 | 0.72 |
| **BDI-II_cr** | 0.226 | 0.15 |

**Abbreviations:** BDI-II, Beck Depression Inventory Second Edition; CINDD, Caregiver’s Inventory Neuropsychological Diagnosis Dementia**;** cr, caregivers; EQ-5D, EuroQol- 5 Dimension (EQ-5D); p, p-value; r, Pearson’s coefficient; RS-14, 14-item Resilience Scale; SD, standard deviation; VAS, Visual Analog Scale.
